# Total Knee Arthroplasty With Long-Stem Implant: A Case Report of a Patient With Extreme Varus Deformity

**DOI:** 10.7759/cureus.67177

**Published:** 2024-08-19

**Authors:** Vishal S Patil, Vinod Nair, Amogh Todkar, Meet Shah

**Affiliations:** 1 Orthopaedics, Dr. D. Y. Patil Medical College, Hospital and Research Centre, Dr. D. Y. Patil Vidyapeeth (Deemed to be University), Pune, IND

**Keywords:** bone defect, varus deformity, arthroplasty, long stem total knee replacement, osteoarthritis

## Abstract

Constrained implants have become more common in difficult primary total knee arthroplasty (TKA) cases in recent years because they may more effectively and conveniently handle the substantial instability that is evident in osteoarthritis of knees with severe varus deformity. However, the need for a constrained TKA in such conditions is controversial, as constraint implants come with a bargain of stability for longitivity. In this case report, we have successfully shown that even in cases of significant instability and bone loss, intraoperative conversion to a restricted device is rarely necessary.

In our case report, a 83-year-old female had complaints of severe pain in bilateral knees, with the right knee more than the left knee, since 12 years with severe varus deformity in the right knee. Physical examination revealed swelling and medial joint line tenderness with restriction of range of motion in bilateral knees. Pre-anesthetic checkup of the patient was done and patient was given clearance for surgery under American Society of Anesthesiologist (ASA)-2, total knee arthroplasty with a long stem was done, extreme varus deformity was corrected, osteophytes removed and tibial bone loss was repaired with bone cement.

Post operatively patient showed significant improvement and McMaster University and Western Ontario Osteoarthritis Index (WOMAC) and Knee Society Scores (KSS) for pain, stiffness, and physical function during everyday activities were significantly improved compared to pre-operative assessment.

## Introduction

Bony deformity, narrowing of the joint space, and degradation of cartilage are the hallmarks of osteoarthritis (OA), which is a degenerative joint disease of the joints. The most effective procedure for elderly patients with severe knee arthritis to reduce pain and disability is total knee arthroplasty (TKA) [1]. Varus deformity is a common complication of advanced OA and can significantly impair gait, function, and quality of life. However, in cases with severe varus deformity and potential bone loss, traditional TKA components may not provide optimal stability and alignment correction. Patients undergoing TKA most commonly have a varus deformity of the affected knee(s). Cartilage and bone loss in the knee joint's medial compartment, together with medial contracture and lateral laxity of ligaments and soft tissues, are characteristics of osteoarthritic varus knees [2]. This case posed many challenges due to severe varus deformity, which included soft tissue balancing, ligament laxity, proximal tibia bone loss, and alignment of the knee joint to restore the knee biomechanics. Long-stem TKA implants can address these challenges by providing additional anchorage in the bone, facilitating deformity correction, and potentially improving long-term outcomes. Constraint implants give stability in varus and valgus knee, but they are reported to have early failure rates. This case report highlights how extreme varus deformity of the knee in osteoarthritis can be operated with conventional long stem implants with proper soft tissue release to give good results.

## Case presentation

**Figure 1:**
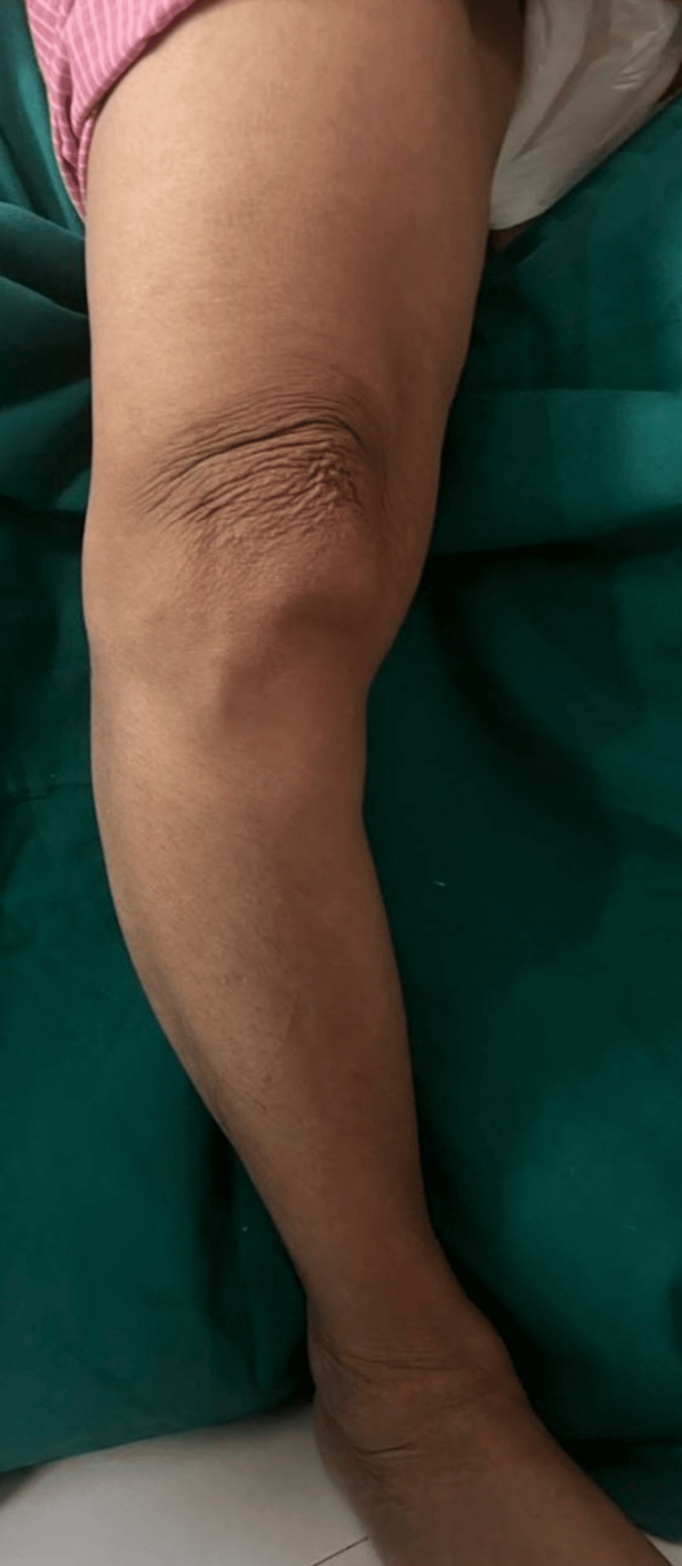
Gross varus deformity of right knee.

An 83-year-old female presented with a 12-year history of right knee pain, progressive worsening over the past year. She reported significant difficulty with walking, stair climbing, cross-legged sitting, and activities of daily living due to pain and stiffness in her right knee. There was no history of fall or trauma or any known history of injury to the right knee. Patient was a known case of hypertension since 10 years, which was controlled on medication. The patient had a gross varus deformity on inspection (Figure 1).

X-rays revealed Kellgren-Lawrence (KL) grade 4 OA with a 35° varus deformity in the right knee and a 20° varus deformity in the left knee. Lateral distal femoral angle (LDFA) on X-ray was calculated to be 100° and medial proximal tibial angle (MPTA) was calculated to be 57° preoperatively. A physical examination confirmed the deformities, limited range of motion in both knees, and significant pain with weight-bearing activities. The McMaster University and Western Ontario Osteoarthritis Index (WOMAC) score was calculated for this patient and turned out to be 76, and the Knee Society Score (KSS) was 10/100, with a Functional KSS score of 35/100, which signifies severe pain, stiffness, and affection for activities of daily living.

**Figure 2:**
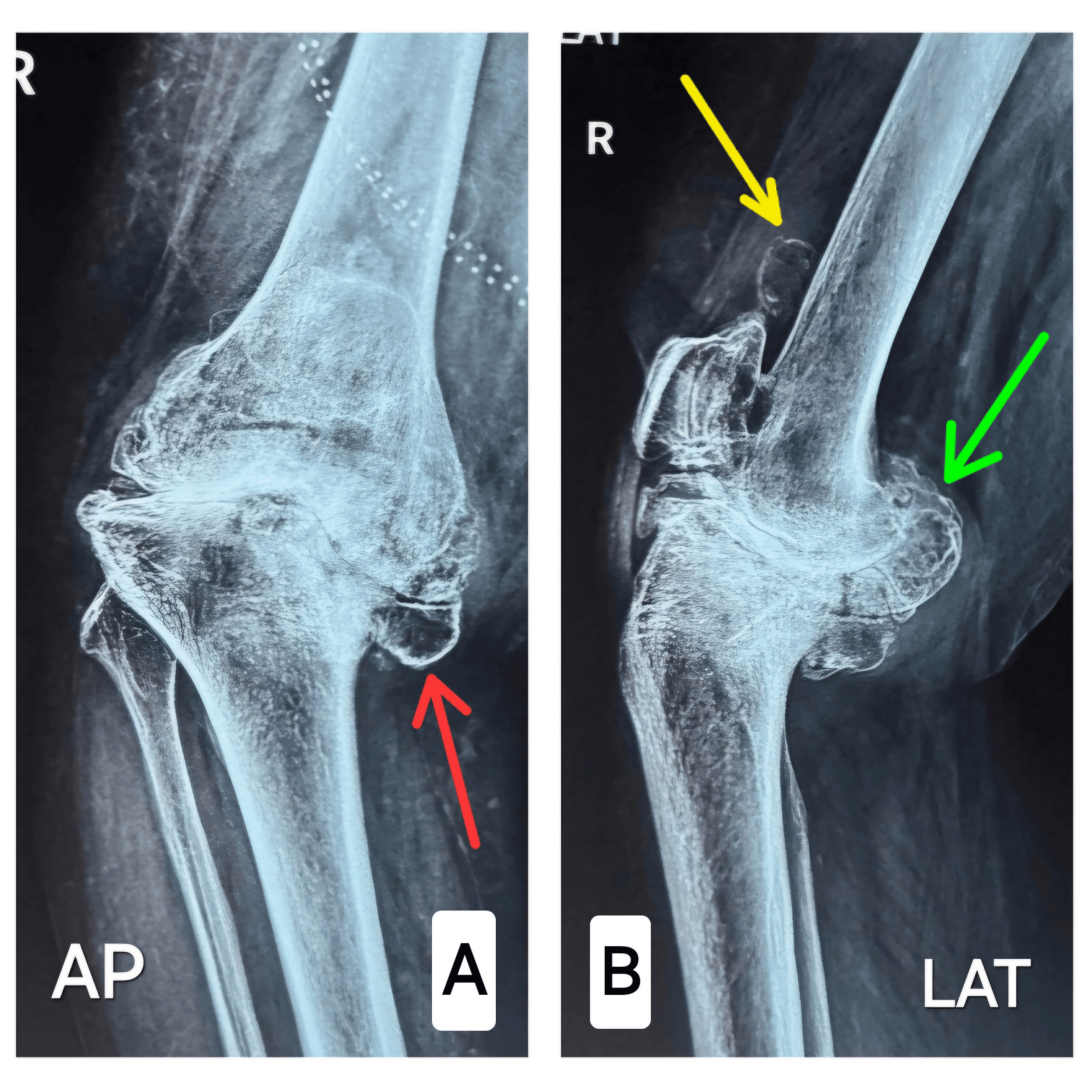
X-ray of right knee showing anteroposterior view and lateral views. A- Red arrow showing proximal tibial bone loss; B- Green arrow showing femoral osteophytes and yellow arrow showing loose body.

**Figure 3:**
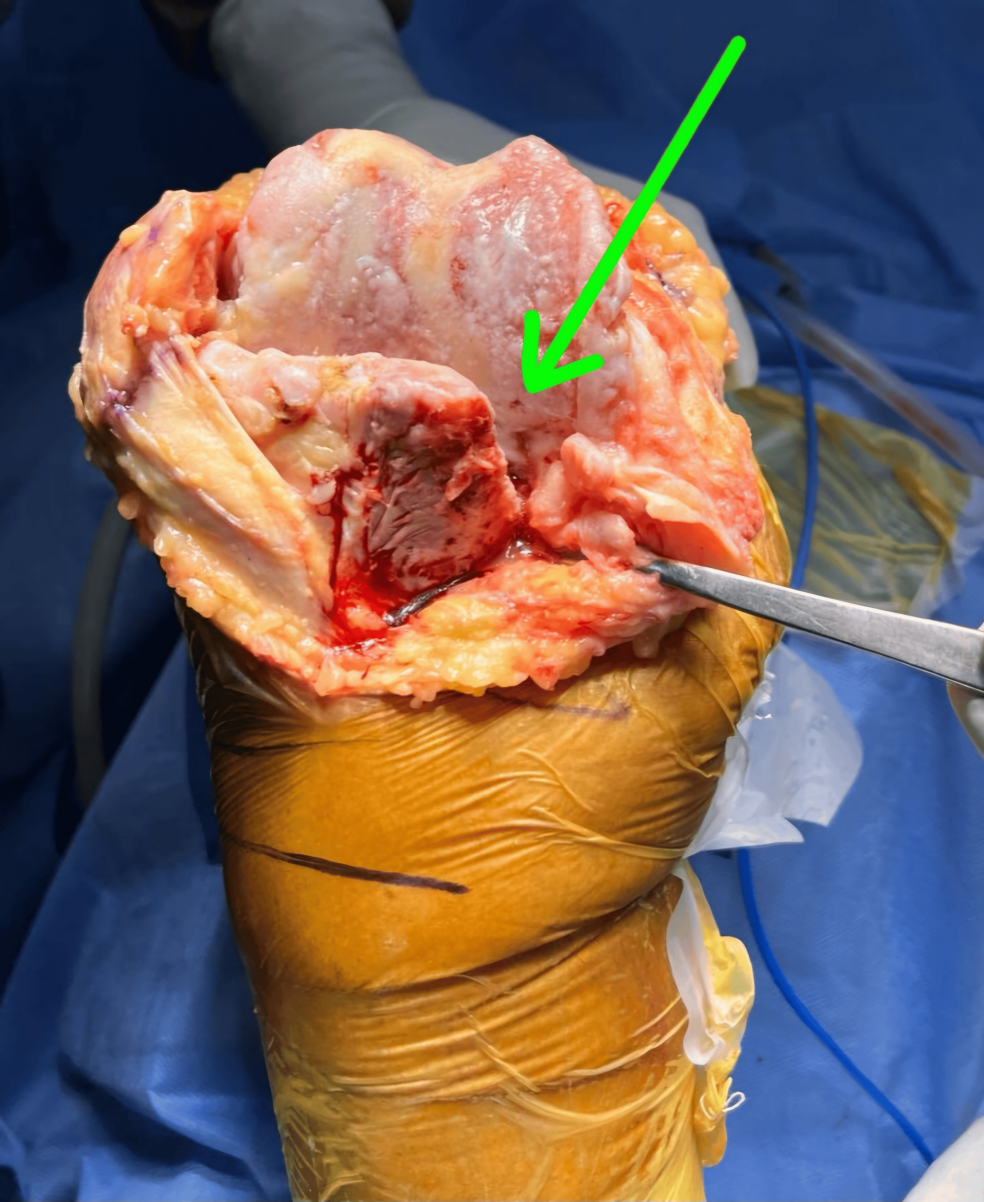
Intraoperative image of surgical exposure of knee joint. Green arrow showing the posteromedial tibial bone loss.

Given the greater severity of the right knee deformity and its greater impact on function, the decision was made to proceed with a cemented right long-stem total knee replacement. A plain radiograph of bilateral knee anterior-posterior view and lateral view (Figure 2) shows right knee involvement with large osteophytes, marked joint space narrowing, and severe sclerosis with proximal tibia posteromedial bone defect (Figure 3).

**Figure 4:**
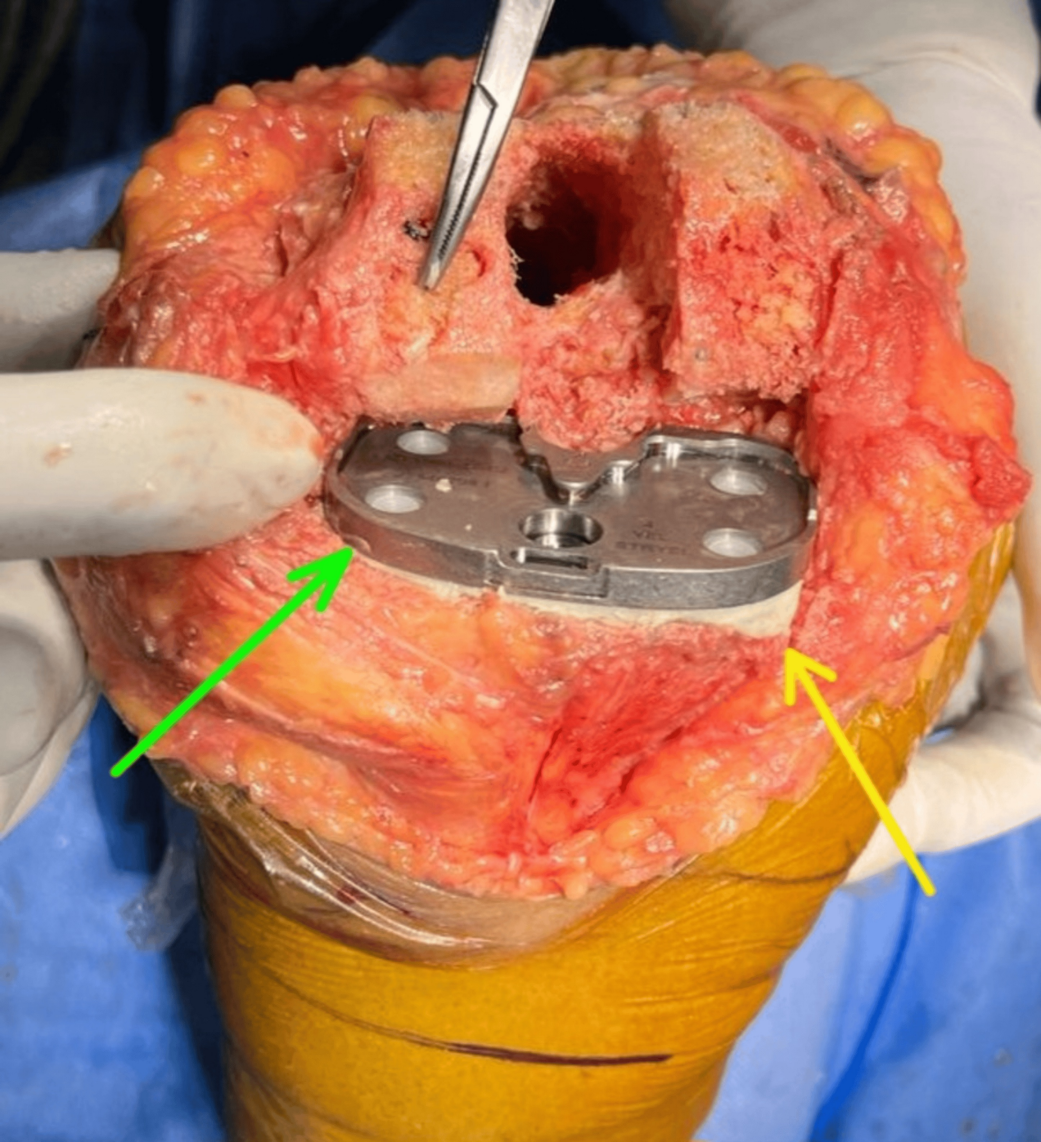
Intraoperative image showing tibial base plate with cement filling. Green arrow showing the tibial base plate; Yellow arrow showing the cement filling in the bone defect.

In this case, the patient was taken in a supine position under spinal with epidural anesthesia with a tourniquet pressure of 280 mmHg. A mid-line rectus splitting approach was taken to expose the right knee. Medial and posteromedial release was done up to pes anserinus and attachment of hamstrings. Medial osteophytes were excised, loose bodies were removed, posterior cruciate ligament was released, and the notch was cleared. Osteophytes on the femoral side below the medial collateral ligament (MCL) were removed, and femoral distal cuts were taken with a 5° intramedullary jig, and a tibial cut was taken with an intramedullary jig 10mm on the lateral side. The extension gap was achieved laterally, and slight tightness on the medial side was present. Femur sizing was done, and cuts were taken at 5° of external rotation. The posterior osteophytes of the femur were removed, and the posterior capsule was released on the medial side. The flexion gap was achieved equally on the medial and lateral sides and checked in extension as well. The implants- the tibial base plate and extension rod- were prepared. Trial was done, and the knee was found to be stable in flexion and extension, and patella tracking was good. Posteromedial bone loss was approximately 1 cm, so two titanium screws of 40 mm were fixed, and the tibial bed was prepared for cementing (Figure 4).

Finally cemented femoral and tibial implants were fixed, and cement was filled in the posteromedial defect. Patella denervation was done with cautery, and the wound was closed in layers. There were no adverse or unanticipated complications intra-operatively or post-operatively. 

**Figure 5:**
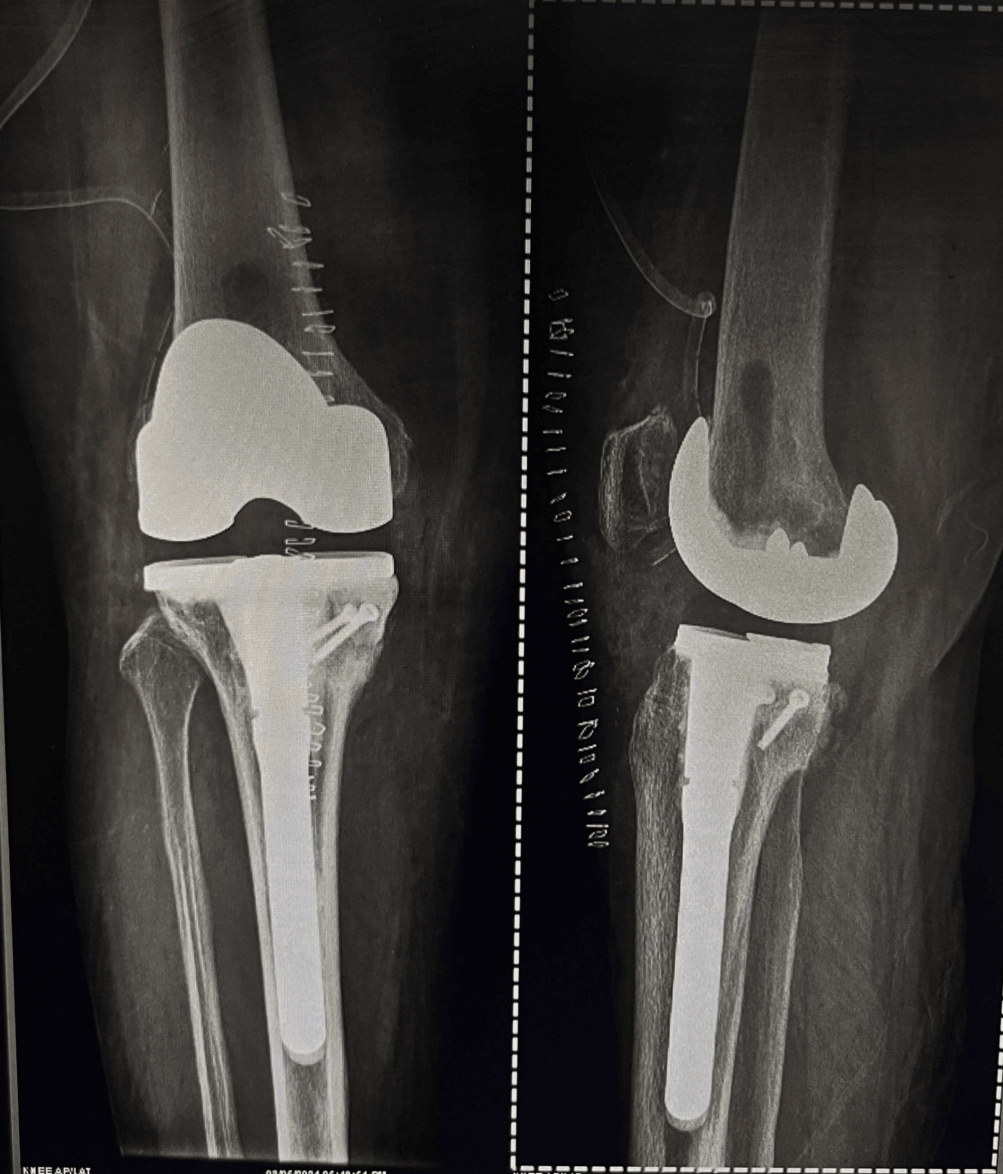
Postoperative X-ray. The extreme varus was corrected, osteophytes cleared on femoral and tibial side, posteromedial bone loss filled with cement and stabilized with screws.

A post-surgery X-ray of the right knee was taken in antero-posterior and lateral views; it shows good implant positioning, correction of the posteromedial defect with cement, good alignment, and deformity correction. LDFA and MPTA post-operative were calculated to be 88° and 90° respectively (Figure 5).

Postoperatively, the patient was started on injection enoxaparin sodium 60 mg subcutaneous till post-op day two and then shifted to oral 10 mg rivaroxaban for three weeks as deep vein thrombosis prophylaxis with deep vein thrombosis stockings for six weeks as per American College of Chest Physicians (ACCP) guidelines for deep vein thrombosis.

The patient was encouraged to do bedside sitting on postoperative day one, and full-weight bearing walking was started on postoperative day two onwards with a walker. The drain was removed on postoperative day two, and sutures were removed on postoperative day 14. The patient followed up in our outpatient department (OPD) at one month, three months and six months postoperatively.

**Figure 6:**
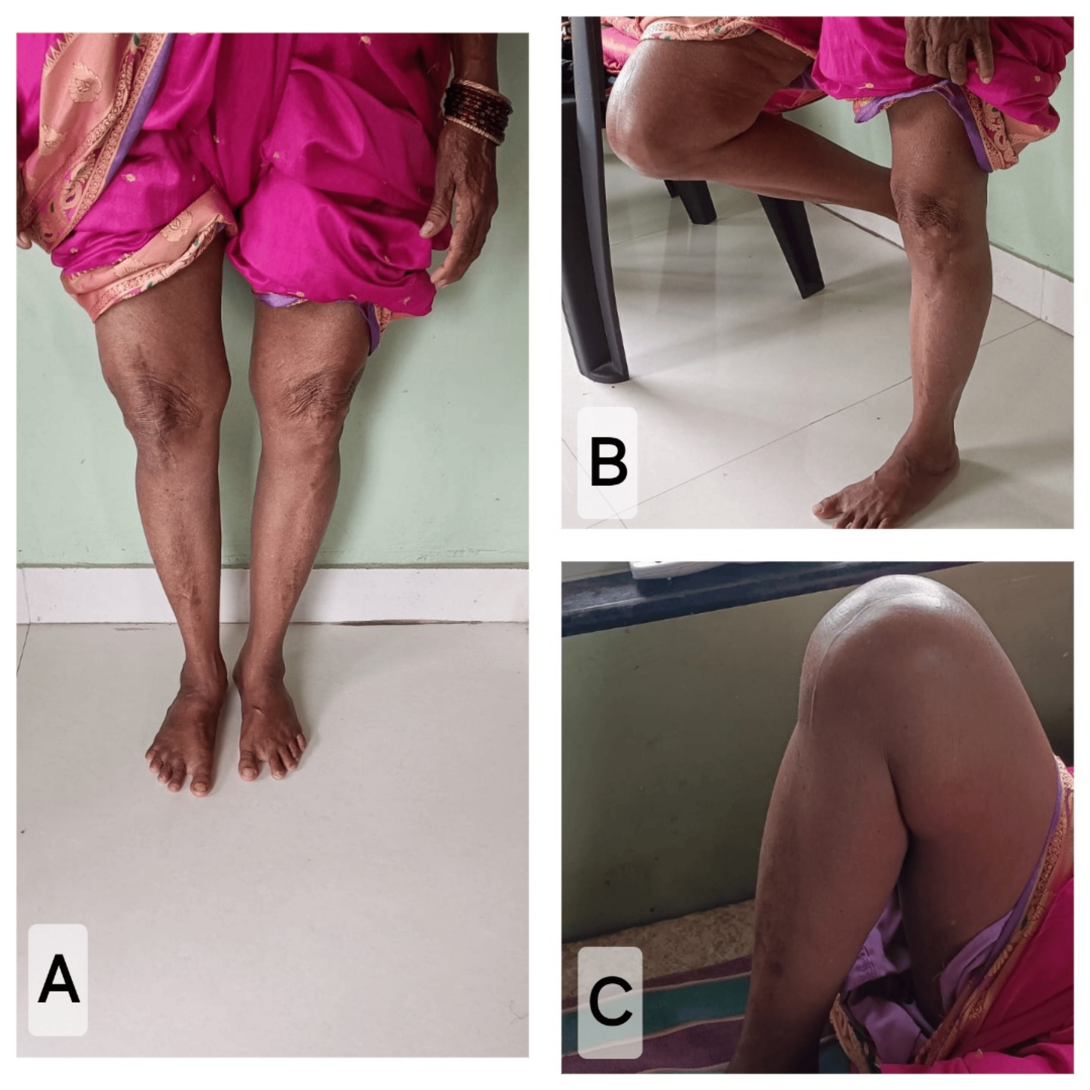
Clinical image of patient at six-month follow-up. A: Clinical image of patient, in standing position with no gross varus deformity. B: Clinical image of patient, demonstrating right knee flexion of 80 degrees, in standing position. C: Clinical image of patient, demonstrating right knee flexion of 110 degrees, in supine position.

Postoperatively, there was considerable improvement in the pain, stability, range of movement of the right knee, alignment of the knee, and activities of daily living. At six-month follow-up the knee society score improved to 85/100, and the functional score improved to 80/100. There was improvement in the WOMAC score as well (Figure 6).

## Discussion

This case demonstrates the successful application of long-stem total knee replacement in managing a complex case of osteoarthritis with severe varus deformity. There are two types of deformities associated with the varus arthritic knee: soft tissue and bone. Lately, Thienpont et al. showed that varus deformity, with a joint line congruency angle of around 3°, is frequently associated with medial tibial disease and lateral joint distraction. An extra-articular deformity needs to be suspected if the varus deformity is more noticeable and the assessed deformity is more significant than the measured intra-articular angles. Femoral bending, also known as varus proximal tibia, is the most prevalent extra-articular deformity in the varus knee [3]. Varus knee also affects soft tissues, which are classified as either dynamic (tendons) or static stabilizers (ligaments). When doing a primary total knee replacement, large posteromedial bone defects are frequently found in the proximal tibia. It is not advised to remove bone down to the level of the defect because, as demonstrated by computed tomography scans, the quantity and quality of the supporting cancellous bone decrease more than 1 centimeter beyond the joint line [4], and furthermore, it is possible for attachments of the patellar ligament, iliotibial band, pes anserinus, and posterior cruciate ligament to become weakened [5]. Injecting cement into the bony defect, cement with screws, bone grafting, customized implants, or metal wedges can all be used to treat the issue of asymmetrical bone loss [6]. As bone loss in our case was less than 1 cm evident on X-ray preoperatively, we did cementing with screw fixation. The defect was very well filled and provided good support to the tibial base plate. In their cadaveric investigation, Rawlinson et al. found that using stem extenders reduces micromotion between the implant and the surrounding bone and increases knee stability [7].

Traditionally, for a successful total knee replacement, rectangular soft tissue balance is very important [8]. Hence, medial soft tissues are often released to achieve proper soft tissue balance in cases of varus deformity [9]. In our case, there was medial tightness, due to which we did MCL release up to attachment of pes anserinus, hamstrings and posterior cruciate ligament and capsule was also released posteriorly. The two most significant static stabilizers are the posterior oblique and superficial medial collateral ligaments. The semimembranosus is the dynamic stabilizer implicated in varus knees. Recently, comparing total knee replacement with a medial stabilizing approach to traditional rectangular gap-balanced total knee replacement, the latter exhibited better postoperative results while minimizing medial soft tissue release and accepting trapezoidal gaps [10]. It is crucial to emphasize that the flexion gap tends to increase more than the extension gap when anterior tissues, such as the superficial medial collateral ligament, are released. In contrast, the gap for extension will be greater than the gap for flexion if additional posterior structures (such as the semimembranosus or posterior oblique ligament) are released. Furthermore, flexion contracture is often associated with a varus deformity of the knee. To increase the flexion gap in posterio-stabilized implants, the posterior cruciate ligament has to be resected [11]. Different classifications have been described for knee deformities. De Muylder et al. have classified them into well-aligned knees (0°-3° deviation), common deformities (4°-10° deviation), substantial deformities (11°-20° deviation), important deformities (21°-30° deviation), and extreme deformities (greater than 30° deviation), which are based on the degree of deformity [12].

Preoperatively, an implant should be chosen on the basis of X-rays and/or computed tomography and clinical evaluation. In cases of minor varus knee deformity (<10°) without flexion deformity, implants like cruciate retaining, postero-stabilized, or medial congruence implants can be considered. In such situations, additional restraint is usually unnecessary because the deformity is typically reducible [13]. In our case, it was associated with flexion contracture, with a fixed flexion deformity of 20°-25° and a 35° varus deformity on X-ray. The posterior cruciate ligament was part of the deformity and was released. In cases of varus deformity, condylar-constrained implants are typically not required. However, a thorough soft tissue release is required whether there is a severe, irreversible varus deformity associated with flexion contracture or not. Furthermore, in cases of severe flexion deformity, semi-constrained implants may be utilized if the knee cannot be properly balanced throughout its range of motion [14].

The long-stem components provided additional stability and facilitated the correction of extreme varus deformity in the right knee. This highlights the potential benefits of this technique in achieving desired alignment and stability with 120 degrees of postoperative flexion of knee (Figure 5), particularly in cases with substantial posteromedial tibial bone loss.

## Conclusions

It is to be concluded that in extreme varus deformity of the knee, if proper step-wise release is done, we can balance the knee without using constraint implants. Bone defects can be reconstructed using cement or augment; in this case, we used cement, and the results were satisfactory. The increasing amount of evidence that supports the use of long-stem total knee replacement for complicated knee osteoarthritis presentations is strengthened by this case report. While an extended period of observation is required to assess the longevity of implants, this case demonstrates the potential for this technique to improve pain, function, and overall quality of life.
